# Adenovirus-associated Deaths in US Military during Postvaccination Period, 1999–2010

**DOI:** 10.3201/eid1803.111238

**Published:** 2012-03

**Authors:** Robert N. Potter, Joyce A. Cantrell, Craig T. Mallak, Joel C. Gaydos

**Affiliations:** Armed Forces Medical Examiner System, Rockville, Maryland, USA (R.N. Potter, J.A. Cantrell, C.T. Mallak);; Armed Forces Health Surveillance Center, Silver Spring, Maryland, USA (J.C. Gaydos)

**Keywords:** adenovirus, vaccine, pneumonia, viral encephalitis, viral pneumonia, public health, molecular epidemiology, viruses, postvaccination period, US military

## Abstract

Adenoviruses are frequent causes of respiratory disease in the US military population. A successful immunization program against adenovirus types 4 and 7 was terminated in 1999. Review of records in the Mortality Surveillance Division, Armed Forces Medical Examiner System, identified 8 deaths attributed to adenovirus infections in service members during 1999–2010.

## Abstract 2

For about 50 years, adenovirus has been a major cause of serious respiratory illness in US active duty military members, particularly at basic training camps. During 1971–1999, a vaccine program successfully lowered the number of illnesses and deaths from adenovirus infection. However, a recent study has shown that since the program ended, the number of deaths might be creeping back up. A new program, which uses second-generation adenovirus vaccines approved in 2011, is expected to again lower the number of illnesses and deaths caused by adenovirus.

Adenoviral respiratory disease has been recognized as a frequent cause of illness in the US active duty military population for >5 decades, particularly at basic training installations (*1**–**5*). A dramatic decrease in adenovirus outbreaks was related to a vaccination program against adenovirus types 4 and 7, which was begun in 1971 (*6**,**7*). After the only manufacturer of the adenovirus vaccines ended production, adenoviral respiratory disease resurged after the phased cessation and eventual termination of adenovirus vaccinations during 1996–1999 (*3**,**4**,**8*). From 1967 through 1998, only 5 adenovirus-associated deaths, all related to types 4 and 7, were reported in active duty military members (*1**,**9**,**10*).

The Mortality Surveillance Division, Armed Forces Medical Examiner System (AFMES), has collected perimortem records for active duty service personnel who died since 1998 (*11*). The Mortality Surveillance Division records were evaluated to identify and describe adenovirus-associated deaths in the US military from 1998 through 2010. Case data and information obtained included age, race, sex, branch of military service, training status, year and location of death, adenovirus type, and clinical features.

## The Patients

During 1998–2010, AFMES recorded ≈18,500 deaths of active duty personnel for all causes. Of these, ≈14,000 were not attributed to combat or hostile action. Of the noncombat, non–hostile action deaths, 121 (0.9%) were caused by confirmed primary infections, including community acquired acute respiratory infections, meningitis, and chronic viral infections, such as hepatitis. Of these, 8 were attributed to adenovirus respiratory disease as the sole contributor or a co-contributor to death after review of available records by an AFMES pathologist (J.A.C.). For these 8 patients, the mean age was 21.3 years (range 18–32 years). Basic demographic data and adenovirus types are shown in the Table. In addition, most decedents were white (6 patients), 1 was black, and 1 was of unknown race. Brief clinical summaries of each case follow.

**Table:** Demographic characteristics and adenovirus type for US military members who died of adenovirus-associated infections, 1998–2010*

Year	Location	Military branch	Age, y/sex	Recruit	Adenovirus type
2000	Illinois	Navy	21/M	Yes	4, 7
2000	Illinois	Navy	18/M	Yes	ND
2003	Missouri	Army	21/M	Yes	4
2003	Oklahoma	Army	18/M	Yes	4, 7
2003	California	Marine Corps	32/M	Yes	4
2004	Oklahoma	Army	22/M	No	ND
2007	Texas	Air Force	19/F	Yes	14
2009	Texas	Air Force	19/M	Yes	14

*ND, not determined.

Patient A had a respiratory infection with adenovirus type 14, which was confirmed by testing of a nasal wash specimen. Several days later he was hospitalized and required care for multilobar pneumonia and acute respiratory distress syndrome. He died 8 days after admission. The autopsy showed necrotizing pneumonia with diffuse alveolar damage. Postmortem lung tissue was positive for adenovirus 14 by PCR.

Patient B was hospitalized with pneumonia 1 month after receiving a diagnosis of infectious mononucleosis. During a hospitalization of 83 days, her course of illness was complicated by multiple bacterial and fungal infections, acute respiratory distress syndrome, pneumothorax, bilateral deep vein thrombosis of lower extremities, acute renal failure, thrombocytopenia, seizures, acute disseminated encephalomyelitis, and acute hemorrhagic leukoencephalitis. PCR testing of serum on admission was positive for adenovirus. Postmortem lung findings included acute bronchopneumonia changes superimposed on diffuse alveolar damage with interstitial chronic inflammation and fibrosis. Postmortem lung tissue was positive for adenovirus 14 by PCR. This case was previously described as part of an adenovirus 14 outbreak (*12*).

Patient C was hospitalized with a 10-day history of treatment for presumed pyelonephritis, extreme weakness, fever, and nausea. He experienced severe sore throat, shortness of breath, chest pain, and mylagias early in the clinical course. Pericardial effusions and pericarditis were identified on the second hospital day. Results of antemortem microbiologic testing were negative, except for a positive serologic test for adenovirus on hospital day 1 with a serum titer of 128 (normal <8). Eighteen days after admission, he died from progressive respiratory failure. Postmortem findings included diffuse alveolar damage, pleural effusions, and fibrinous pericarditis. No postmortem microbiologic testing was done. The autopsy did not indicate a specific cause of death.

Patient D was treated for pneumonia as an outpatient. Two days later, he was found dead. Results of antemortem microbiologic testing were negative. Postmortem findings included pulmonary edema, congestion, and chronic inflammatory cell infiltrates with pleural effusions. Postmortem lung tissue was positive for adenovirus 4 by PCR.

Patient E was hospitalized and treated for pneumonia and died ≈25 days after admission. The autopsy showed pneumonia with superimposed fungal infection (consistent with aspergillosis) and septic emboli to the heart, brain, liver, spleen, gastrointestinal tract, and kidneys. Autopsy lung tissue was positive by PCR for adenovirus types 4 and 7.

Patient F became unresponsive during training and could not be resuscitated; his temperature was 105.5°F. He had sought treatment 1 week earlier for a presumed viral respiratory illness. The autopsy showed multilobar pneumonia. Lung tissue was PCR positive for adenovirus 4. *Neisseria meningitidis* was also identified by PCR and culture of lung tissue.

Patient G was found unresponsive on day 4 of outpatient treatment for a presumed upper respiratory infection. He was hospitalized and died 11 days later. Antemortem microbiologic testing results were negative. A complete autopsy was not performed, but selected specimens were obtained. A postmortem brain biopsy specimen showed histologic changes consistent with viral encephalitis. Brain and lung tissue were positive by PCR for adenovirus (*13*). Serum specimens had antibodies to adenovirus types 4 and 7 (*13*).

Patient H sought treatment as an outpatient for cough, shortness of breath, and fever of several days’ duration. The next day he returned with severe dyspnea, weakness, and a petechial rash. Patient H was noted to be in acute multiorgan failure and died within 12 hours. Antemortem testing was negative for microbiologic agents. The autopsy showed diffuse hemorrhagic pneumonia and diffuse alveolar damage. Results of postmortem viral and bacterial cultures were negative. By report, PCR of postmortem lung tissue was positive for adenovirus. This person also met the criteria for group A streptococcal toxic shock syndrome (*13*).

Of these 8 patients, only 3 had an adenovirus infection identified before death. The remaining 5 patients received a diagnosis on the basis of postmortem tissue examination. None had documentation of adenovirus vaccination.

## Conclusions

Eight deaths among members of the US military were attributed to adenoviral respiratory disease by an AFMES pathologist during 1998–2010. All 8 deaths occurred after the adenovirus types 4 and 7 immunization program ended in 1999. Five earlier adenovirus-associated deaths in US military service members have been identified and documented. These occurred in 1967 (1 death), 1972 (3 deaths), and 1974 (1 death) (*1**,**9**,**10*). We did not identify any adenovirus-associated deaths in the US military during 1975–1998. Differences in medical surveillance and laboratory capabilities preclude attempting to make meaningful comparisons of the risk for adenovirus-associated death during the prevaccine, vaccine, and postvaccine periods.

The population at greatest risk for adenovirus-associated disease is military recruits. Therefore, the findings shown in the Table are not surprising. Most recruits are young men, and the deaths reported here occurred at the recruits’ training centers. Second-generation live oral vaccines against adenovirus types 4 and 7 were approved by the US Food and Drug Administration in March 2011 (*14**,**15*). After a 12-year absence, the adenovirus vaccination program for military recruits resumed in October 2011. Surveillance of the recruit centers will continue for evaluation of the types 4 and 7 vaccines. The military medical community is hopeful that the protective effect of the vaccines will extend beyond adenovirus types 4 and 7.

## References

[1] Dudding BA, Wagner SC, Zeller JA, Gmelich JT, French GR, Top FH Jr. Fatal pneumonia associated with adenovirus type 7 in three military trainees. N Engl J Med. 1972;286:1289–92. 10.1056/NEJM197206152862403 4337012

[2] Ludwig SL, Brundage JF, Kelley PW, Nang R, Towle C, Schnurr DP, Prevalence of antibodies to adenovirus serotypes 4 and 7 among unimmunized US Army trainees: results of a retrospective nationwide seroprevalence survey. J Infect Dis. 1998;178:1776–8. 10.1086/314498 9815232

[3] Barraza EM, Ludwig SL, Gaydos JC, Brundage JF. Reemergence of adenovirus type 4 acute respiratory disease in military trainees: report of an outbreak during a lapse in vaccination. J Infect Dis. 1999;179:1531–3. 10.1086/314772 10228076

[4] Gray GC, Goswami PR, Malasig MD, Hawksworth AW, Trump DH, Ryan MA, ; Adenovirus Surveillance Group. Adult adenovirus infections: loss of orphaned vaccines precipitates military respiratory disease epidemics. Clin Infect Dis. 2000;31:663–70. 10.1086/313999 11017812

[5] Russell KL, Broderick MP, Franklin SE, Blyn LB, Freed NE, Moradi E, Transmission dynamics and prospective environmental sampling of adenovirus in a military recruit setting. J Infect Dis. 2006;194:877–85. 10.1086/507426 16960774 PMC7109706

[6] Top FH Jr. Control of adenovirus acute respiratory disease in US Army trainees. Yale J Biol Med. 1975;48:185–95. 1099823 PMC2595226

[7] Gaydos CA, Gaydos JC. Adenovirus vaccines in the U.S. military. Mil Med. 1995;160:300–4. 7659229

[8] McNeill KM, Hendrix RM, Lindner JL, Benton FR, Monteith SC, Tuchscherer MA, Large, persistent epidemic of adenovirus type 4-associated acute respiratory disease in US army trainees. Emerg Infect Dis. 1999;5:798–801. 10.3201/eid0506.990609 10603214 PMC2640815

[9] Levin S, Dietrich J, Guillory J. Fatal nonbacterial pneumonia associated with adenovirus type 4. Occurrence in an adult. JAMA. 1967;201:975–7. 10.1001/jama.1967.03130120083029 4293673

[10] Loker EF Jr, Hodges GR, Kelly DJ. Fatal adenovirus pneumonia in a young adult associated with ADV-7 vaccine administered 15 days earlier. Chest. 1974;66:197–9. 10.1378/chest.66.2.197 4368721

[11] Gardner JW, Cozzini CB, Kelley PW, Kark JA, Peterson MR, Gackstetter GD, The Department of Defense Medical Mortality Registry. Mil Med. 2000;165(Suppl 2):57–61. 10920642

[12] Brosch L, Tchandja J, Marconi V, Rasnake M, Prakash V, McKnight T, Adenovirus serotype 14 pneumonia at a basic military training site in the United States, spring 2007: a case series. Mil Med. 2009;174:1295–9. 20055071 10.7205/milmed-d-03-0208

[13] Centers for Disease Control and Prevention. Two fatal cases of adenovirus-related illness in previously healthy young adults—Illinois, 2000. MMWR Morb Mortal Wkly Rep. 2001;50:553–5. 11456329

[14] Lyons A, Longfield J, Kuschner R, Straight T, Binn L, Seriwatana J, A double-blind, placebo-controlled study of the safety and immunogenicity of live, oral type 4 and type 7 adenovirus vaccines in adults. Vaccine. 2008;26:2890–8. 10.1016/j.vaccine.2008.03.037 18448211

[15] US Food and Drug Administration, US Department of Health and Human Services. Vaccines, blood, and biologics. March 16, 2011 approval letter—adenovirus type 4 and type 7 vaccine, live, oral [cited 2011 Jul 7]. http://www.fda.gov/BiologicsBloodVaccines/Vaccines/ApprovedProducts/ucm247511.htm

